# Tanner–Whitehouse skeletal maturity score derived from ultrasound images to evaluate bone age

**DOI:** 10.1007/s00330-022-09285-2

**Published:** 2022-12-03

**Authors:** Pin Lv, Chao Zhang

**Affiliations:** Department of Medical Ultrasound, Tongji Hospital of Tongji Medical College of Huazhong University of Science and Technology, 1095 Jiefang Rd, Wuhan, 430030 China

**Keywords:** Bone age, Ultrasound, Radiography, Ossification

## Abstract

**Objective:**

The complexity of radiographic Tanner–Whitehouse method makes it less acceptable by radiologists and endocrinologists to assess bone age. Conventional ultrasound could be used to measure the ratio of the height of the ossification center to the epiphysis of the bone to evaluate maturity of bone. The purpose of this study is to obtain radiographic TW3 skeletal maturity score with ultrasound images.

**Methods:**

In this prospective diagnostic study, participants aged between 1 and 18 years undergoing radiography for bone age evaluation were evaluated from April 2019 to November 2021. Ultrasonic skeletal maturity scores of participants were transformed into radiographic skeletal maturity scores with the fitted formulas established in this study. Diagnostic performances of the transformed scores to diagnose advanced or delayed bone age were confirmed. Ultrasound images of 50 participants in the validation group were re-evaluated to confirm inter-rater reliability.

**Results:**

A total of 442 participants (median age, 9.5 years [interquartile range, 7.8–11.1 years]; 185 boys) were enrolled. Ultrasound determination of bone age had a sensitivity of 97% (34/35, 95% CI: 83, 99) and a specificity of 98% (106/108, 95% CI: 93, 99) to diagnose advanced or delayed bone age. The intra-class correlation coefficient for inter-rater reliability was 0.993 [95% CI: 0.988, 0.996], *p* < 0.0001.

**Conclusions:**

Radiographic Tanner–Whitehouse skeletal maturity score could be obtained from ultrasound images in a simple, fast, accurate, and radiation-free manner.

**Key Points:**

*• The fitting formulas between radiographic TW3 skeletal maturity score and ultrasonic skeletal maturity score were developed.*

*• Through measurement of ossification ratios of bones with ultrasound, TW3 skeletal maturity score was obtained in a simple, fast, and radiation-free manner.*

## Introduction

Bone age is an index to evaluate skeletal maturity in children [1, 2]. The hand and wrist radiographs interpreted by the Greulich–Pyle (GP) atlas [3] and the Tanner–Whitehouse (TW2 and TW3, the second and the third edition) method [4, 5] are the most commonly used to assess bone age [6]. The GP atlas, used by about 76% of the radiologists or endocrinologists [7], is easy to learn but it is more reviewer-dependent [2]. Furthermore, the standard hand images in the GP atlas were collected in upper-class Caucasian children. Ethnic and racial differences in growth patterns restricted the use of GP method in Asian [8], African [9], and children in other ethnic groups [2, 10]. The TW method may be more accurate [10, 11], but it is more complicated and time consuming [12]. Artificial intelligence models have been used to interpret radiographs in order to alleviate intra-or inter-variability in bone age evaluation. But, they are still in an early phase of development [13–16]. What’s more, the X-ray machine may not be available for as much as three-quarters of the world’s population [17].

Ultrasound is non-ionizing, becoming cheaper and more portable [18, 19]. Recent studies by Wan et al [18, 20, 21] focused on evaluation of bone age by conventional ultrasound. Ossification ratio, defined as the ratio of the height of the ossification center to the epiphysis of the bone, was calculated with conventional ultrasound to evaluate skeletal maturity. The authors considered bone maturation as a process of the ossification ratio from 0 to 100% [20]. The ultrasonic skeletal maturity score (SMS), the summation of ossification ratios of the radius, ulna, and femur multiplied by 100, was used to evaluate bone age [17, 21]. The study [21] confirmed that with such scoring system, conventional ultrasound could help diagnose abnormal bone age with high sensitivity (93% for boys, 100% for girls) and specificity (98% for boys, 98% for girls) in Chinese children. But, to our knowledge, no reference standard of such ultrasonic scoring system is applicable to other ethnic groups. Both ultrasonic SMS [21] and TW SMS [22] are quantitative description of maturity of bones. The fundamental data are independent of ethnic group [22]. Now that the percentile charts for TW3 SMS have been established in more than one ethnic groups [5, 22–24], we hypothesized that after the connection between the two kinds of SMSs being established, the TW3 SMS of a patient could be obtained from his or her ultrasound images, instead of an X-ray film, to evaluate bone age by checking percentile charts for the corresponding ethnic groups in a simple and radiation-free manner.

The purpose of this study is to obtain radiographic TW3 SMS from ultrasound images and to determine the reliability of the ultrasonic TW3 SMS (USTW3 SMS) to assess bone age.

## Methods

### Patients

**Fig. 1:**
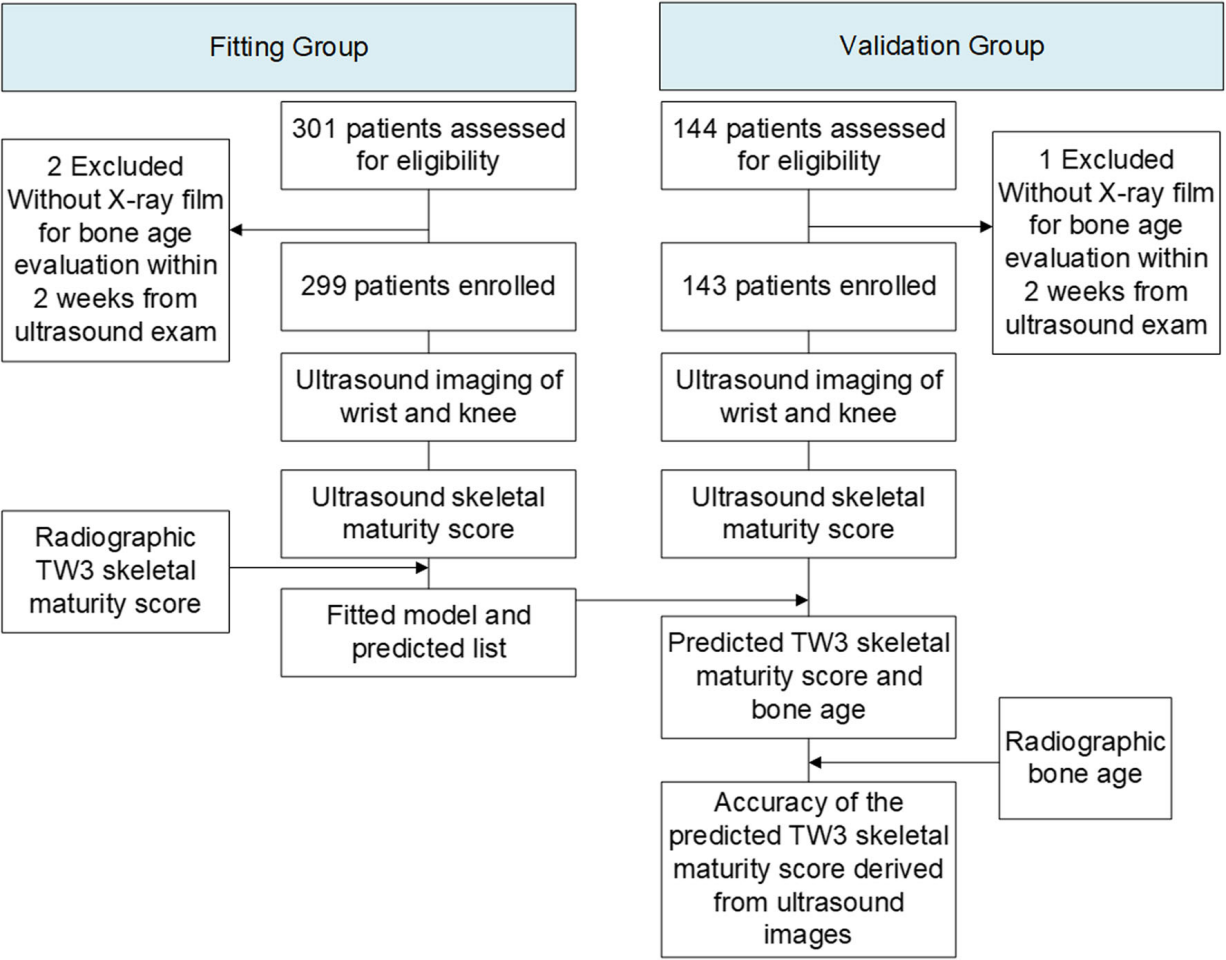
Flow chart of the study

Our prospective diagnostic accuracy study followed Standards for the Reporting of Diagnostic Accuracy Studies guidelines [25]. It was approved by the ethics committee of our hospital and registered on the Chinese clinical trial registry website (http://www.chictr.org.cn, number ChiCTR1900027917). The patients were enrolled from the pediatric outpatient service in our hospital from April 2019 to November 2021. Informed consents from guardians of the patients were obtained. Inclusion criteria were Chinese children aged between 1 year and 18 years. Patients who did not undergo radiography for bone age evaluation within 2 weeks after ultrasound evaluation were excluded. The patients were divided into a fitting group and a validation group according to enrolling time and sample size calculation (Fig. 1).

### Equipment and imaging protocol

Ultrasound imaging was performed with a Philips EPIQ 7 system (Philips) equipped with a linear array eL18-4 transducer by one operator who has evaluated hundreds of bone age ultrasound examinations according to the protocol described by Wan et al. [21] Briefly, the ultrasonic probe was placed longitudinally oriented along the distal lateral aspects of radius and ulna to image styloid process of these two bones. The probe was placed along the medial collateral ligament to image the medial epicondyle of femur. The ultrasound images were archived for further analysis. The time taken to obtain images was recorded. The maximum height of ossification center and epiphysis of bones were measured twice by the same radiologist to obtain ossification ratio. The ossification ratios of the radius, ulna, and femur of the patient were summed and then multiplied by 100 to obtain ultrasonic SMS. The time taken to calculate SMS was recorded. To analyze inter-rater repeatability, ultrasonic images from 50 patients were re-evaluated by another radiologist. All the radiologists were blinded to the medical information of the patients.

Radiographs of left hand and wrist of the patients were interpreted with the TW3-RUS method for the Chinese population [23] by two radiologists who were blinded to the patients’ medical information. When different TW3 SMSs were evaluated by the two radiologists, the mean value was used to determine the TW3 SMS of the patient. Radiographs of patients in the validation group were evaluated with TW3 method by the same two radiologists.

### Curve fitting between ultrasonic SMS and radiographic TW3 SMS

The correlation between ultrasonic SMS and TW3 SMS of patients in the fitting group was analyzed. Simple linear regression, polynomial regression, Box–Cox power exponential distribution, Box–Cox Cole and Green distribution, Box–Cox t distribution, and normal distribution were tested for curve fitting. The distribution and the corresponding formulas with the smallest generalized Akaike information criterion, or GAIC [3], value were obtained for optimal fit. Worm plots and Z statistics were used to test goodness of fit [26].

### Transformation of ultrasonic SMS into TW3 SMS

The corresponding formulas were used to obtain the USTW3 SMS of the patients in the validation group based on their ultrasonic SMS. The agreement between the USTW3 SMS transformed from ultrasound images and the TW3 SMS obtained from radiographs of the patients was analyzed.

### Diagnostic performances

Both the USTW3 SMS and the radiographic TW3 SMS were used to evaluate bone age of patients according to the TW3 chart for Chinese children [23]. Agreements between the two kinds of bone ages were analyzed. Sensitivity and specificity of the USTW3 SMS to diagnose advanced or delayed bone age were determined using radiographic bone age as the reference standard. SMSs between the 2.5th percentile and 97.5th percentile were considered normal, while SMSs less than the 2.5th percentile or greater than the 97.5th percentile were considered delayed or advanced, respectively [2, 27].

The absolute difference between the bone ages evaluated by USTW3 and by the reference standard method (TW3) was calculated for the patients in the validation group.

### Statistical analysis

Sample size calculations were made a priori for diagnostic test. The sample size calculations assumed an *α* of 0.05, under 2-sided hypothesis testing, and *β* error of 0.10. Radiographic TW3 SMSs for ultrasonic SMS curves were fitted with the generalized additive model for location, scale, and shape package, or GAMLSS, in R 3.6.3 (The R Foundation for Statistical Computing). Statistical analyses were conducted using SPSS 22^.^0 (IBM) and Prism 8^.^0 (GraphPad). The differences between the USTW3 SMS and the radiographic TW3 SMS were analyzed by a paired samples *t* test. The agreements between the USTW3 SMS and the radiographic TW3 SMS were analyzed by Bland–Altman analysis. The differences and agreements between bone ages evaluated by different methods were analyzed with the same method. The comparisons for categorical variables were performed by a *X* [2] test. Intra-class correlation coefficient was calculated to confirm inter-rater reliability. Sensitivity and specificity were used to estimate the diagnostic performance. All statistical tests were 2 sided with a *p* value < 0.05 considered significant.

## Results

A total of 442 patients (median age, 9.5 years [interquartile range, 7.8–11.1 years]; 185 boys) were enrolled, including 299 patients (median age, 9.5 years [interquartile range, 7.8–11.3 years]; 125 boys) in the fitting group and 143 patients (median age, 9.4 years [interquartile range, 7.7–10.8 years]; 60 boys) in the validation group. More characteristics of the patients are listed in Table 1.

**Table 1:** Patient characteristics

	Fitting group		Validation group	
	Boys	Girls	Boys	Girls
Number	125	174	60	83
Age (y)*	12.5 (8.0–11.0)	9.1 (7.8–10.3)	10.8 (9.1–11.6)	8.6 (7.3–9.9)
Complain				
Precocious puberty	24	100	13	56
Short stature	41	29	27	12
Obesity	22	7	9	5
Hyperthyroidism	3	1	-	4
Adrenal cortical hyperplasia	3	-	5	-
Small penis	4	-	2	-
Hypothyroidism	2	-	-	2
Adrenocortical insufficiency	1	-	1	-
Physical checkup	25	37	3	4

Note. *Data were median (interquartile range) chronologic age

**Fig. 2:**
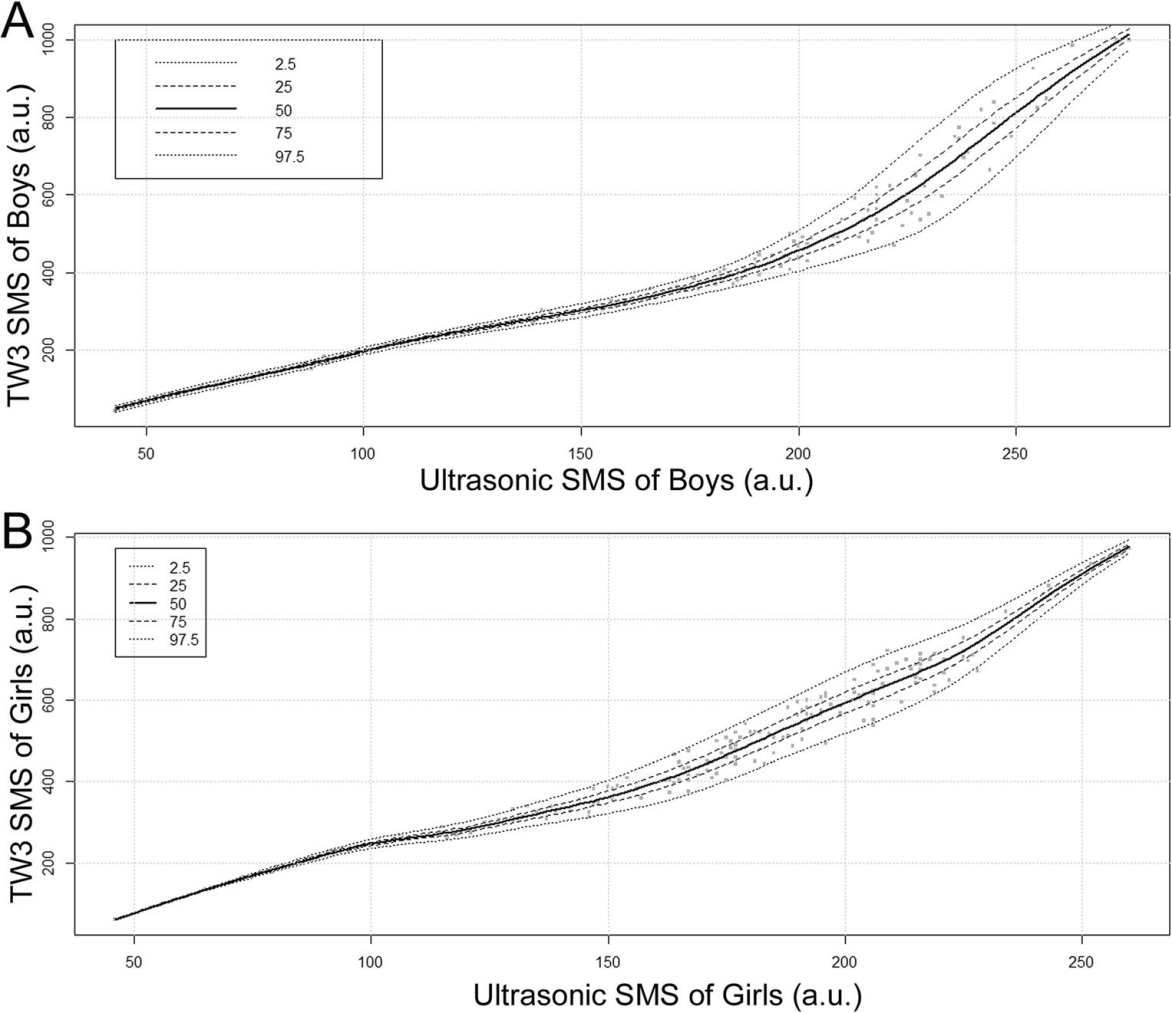
Percentile fitting curves for TW3 skeletal maturity score for ultrasonic skeletal maturity score (SMS) of (**A**) boys and (**B**) girls

Normal distribution and the smoothing function P-splines were confirmed the best for curve fitting of radiographic TW3 SMS for ultrasonic SMS. For boys, the selected formula was as follows: NORMAL [(λ = 1.5, df(μ) = 8.8, df(σ) = 5.7]; for girls, NORMAL [(λ = 1.3, df(μ) = 9.6, df(σ) = 4.9], where λ is the power of the transformation applied to ultrasonic SMS before fitting the model, df(μ) the degree of freedom for fitting the median, and df(σ) the degree of freedom for fitting the coefficient of variation. The fitting curves are shown in Fig. 2. The worm plots and Z statistics for the selected models implied that the fit was adequate [25]. The 50th percentile values of USTW3 SMS for ultrasonic SMS predicted by the fitted formulas are listed in Table 2.

**Table 2:** Predicted TW3 skeletal maturity score (SMS) for ultrasonic SMS (excerpt)

Ultrasonic SMS	Predicted TW3 SMS (boys)	Predicted TW3 SMS (girls)
155	312	379
156	315	383
157	317	386
158	319	390
159	321	394
160	324	398
161	326	401
162	329	405
163	331	410

**Fig. 3:**
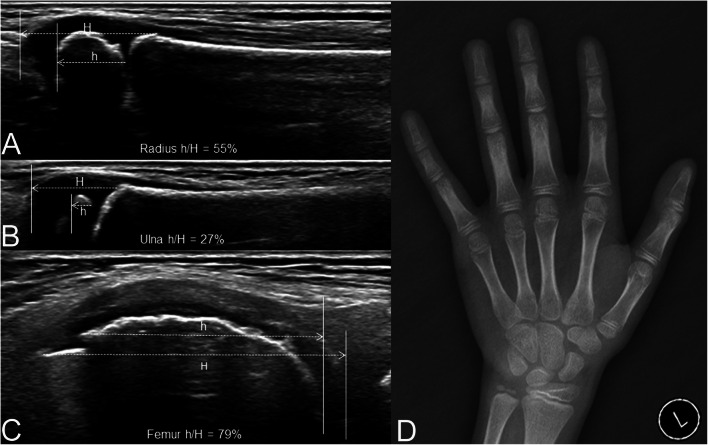
Ultrasound images of the distal end of the left radius (**A**), ulna (**B**), and femur (**C**) and the X-ray film of left hand and wrist (**D**) in a 10.9-year-old Chinese boy with complaint of precocious puberty

The USTW3 SMSs of the patients in the validation group were derived from their ultrasonic SMS by checking the lists in Table 2. For example, ultrasound images and the X-ray film of left hand and wrist in a 10.9-year-old Chinese boy with complaint of precocious puberty are shown in Fig. 3. The ultrasonic SMS was calculated as the summation of the ossification ratios (noted as h/H in ultrasound images) of radius, ulna, and femur multiplied by 100, i.e., (55% + 27% + 79%)*100 = 161. The ultrasonic SMS was then transformed into USTW3 SMS, which was 326, by checking Table 2. The radiographic SMS derived from the X-ray film by TW3 method was 325. After checking the list of TW3 SMS-for-age for Chinese boys [23], the bone age of the patient derived from ultrasound images and from X-ray film was confirmed to be the same, both 10.3 years.

No differences between the USTW3 SMS and TW3 SMS were observed (0.4, 95% CI: −5.3, 6.1; *p* = .88). The 95% limits of agreement of the USTW3 SMS and the TW3 SMS were −67.1 (95% CI: −76.6, −57.4) to 67.9 (95% CI: 58.3, 77.6).

**Fig. 4:**
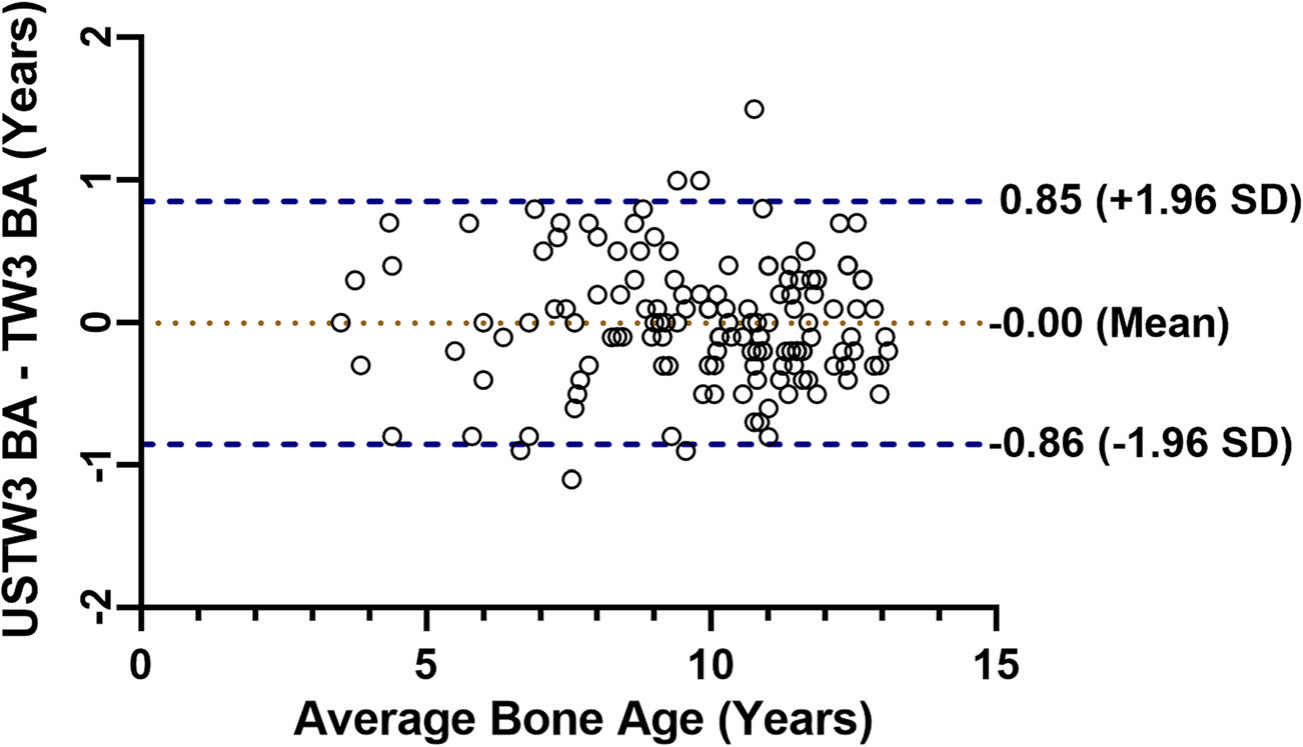
Bland–Altman plot of the difference versus average age between bone ages evaluated with transformed ultrasonic Tanner–Whitehouse (USTW3) method and with radiographic TW3 method. SD, standard deviation

No differences between the USTW3 bone age and TW3 bone age were observed (−0.001 year, 95% CI: −0.07, 0.07 year; *p* = 0.97). The agreement of the USTW3 bone age and the TW3 bone age was shown with Bland–Altman plots (Fig. 4). The 95% limits of agreement of the USTW3 bone age and the TW3 bone age were −0.86 (95% CI: −0.98, −0.73) to 0.85 (95% CI: 0.73, 0.98) years.

With radiographic bone age as a reference standard, the transformed USTW3 SMS had a sensitivity of 97% (34/35, 95% CI: 83, 99) and a specificity of 98% (106/108, 95% CI: 93, 99) for diagnosing advanced or delayed bone age.

The inter-rater repeatability of bone age derived from the USTW3 SMSs measured by two investigators was high (the intra-class correlation coefficient was 0.993 [95% CI: 0.988, 0.996], *p* < .001). 95% limits of agreement of inter-rater variation were −0.40 (95% CI: −0.50, −0.31) to 0.40 (95% CI: 0.31, 0.50) years.

The mean time ± standard deviation for ultrasound images acquisition was 2 min ± 2. The mean time ± standard deviation for calculation of ultrasonic SMS was 1 min ± 1. The ultrasonic SMS could be transformed into USTW3 SMS immediately by checking the relevant lists. The overall time of the whole process, from the beginning of ultrasound examination to the acquirement of bone age in the end, was 3 min ± 2.

## Discussion

Radiographic GP method for bone age evaluation is easy to learn but less generalizable to children of other ethnicities except for white population [2, 28]. Radiographic TW3 method has been used in different races [22, 23], but the complexity of the method makes it less acceptable by radiologists and endocrinologists. Conventional ultrasound has been used to evaluate bone age in Chinese, but not in other ethnic groups [21]. In this study, radiographic TW3 SMS was derived from ultrasonic SMS by the fitting formulas to evaluate bone age.

No difference of the value was found between the transformed USTW3 bone age and the TW3 bone age. The USTW3 SMS could be used to diagnose advanced or delayed bone age with high sensitivity and specificity. These indicate the accuracy of the USTW3 bone age was high using TW3 bone age as a reference standard.

Bull et al [29] showed the intra-observer variation (95% limits of agreement) was −2.46 to 2.18 years for the GP method and −1.41 to 1.43 years for the TW2 method. Yildiz et al [30] showed the intra-observer variation (95% limits of agreement) for the GP method and TW2 method was −0.77 to 0.97 and −0.45 to 0.37, respectively. In our study, the inter-observer variation years were smaller. This indicates the higher repeatability of the USTW3 method invented in our study compared to the aforementioned studies.

King et al [31] gave the average time taken was 7.9 min for TW2 and 1.4 min for GP assessments. In our study, the average time taken for USTW3 assessment, i.e., calculating ultrasonic SMS and then transformed into TW3 SMS, was 1 min. The complicated and time-consuming process to obtain TW3 SMS has been much more simplified by the modality established in our study. Ultrasound is at least an auxiliary method to radiography in evaluating bone age. The radiation-free nature of ultrasound may make it more accessible to patients and guardians.

There are some issues to be addressed in our study. First, the sample size for toddlers and near adults in the fitting group was small. This may result in risks of increasing error for predicting USTW3 SMS for children of these ages. Second, the inter-rater reliability was based on the ultrasound images scanned by one operator. It is known that ultrasound is an operator-dependent imaging modality. The standard scanning protocol and specific training to the operator may increase the inter-operator reliability and need further study. Third, the participants were from a single race. Nevertheless, both ultrasonic SMS and TW3 SMS are description of development of bones. They are anthropometric measurements, comparable with height or weight, independent of ethnic group [22]. The fitting formulas for transformation of SMSs have the potential to be applied in other ethnic groups.

In summary, radiographic Tanner–Whitehouse skeletal maturity score could be obtained from ultrasound images in a simple, fast, accurate, and radiation-free manner. The transformed skeletal maturity score could be used to assess bone age with high reliability.

## Abbreviations

GP: Greulich–Pyle
SMS: Skeletal maturity score
TW: Tanner–Whitehouse
USTW3 SMS: Ultrasonic TW3 SMS

## Declarations

### Guarantor

The scientific guarantor of this publication is Chao Zhang.

### Statistics and biometry

No complex statistical methods were necessary for this paper.

### Methodology

• prospective

• diagnostic or prognostic study

• performed at one institution
